# Visualizing a marker’s degrees of necessity and of sufficiency in the predictiveness curve

**DOI:** 10.1186/s12874-025-02544-y

**Published:** 2025-04-23

**Authors:** Andreas Gleiss

**Affiliations:** Center for Medical Data Science, Institute of Clinical Biometrics, Medical University of Vienna, Spitalgasse 23, 1090 Vienna, Austria

**Keywords:** Predictiveness curve, Necessity, Sufficiency, Explained variation

## Abstract

**Background:**

The degrees to which a factor is necessary or sufficient for an event have been proposed as generalizations of attributable risk based on simple functions of unconditional and conditional event probabilities. Predictiveness curves show the risk for an event, as derived by a model with one or more predictors, depending on risk percentiles that represent the predictors’ distribution in the underlying population.

**Methods:**

Connections between the degrees of necessity and of sufficiency and explained variation on the one hand and the predictiveness curve on the other hand are mathematically proved and exemplified using data of in-hospital death of Covid- 19 patients.

**Results:**

We show that the degrees of necessity and of sufficiency can be represented as proportions of areas easily identifiable in the plot of the predictiveness curve. In addition, we show that the proportion of explained variation, a common measure of predictiveness and relative importance of prognostic factors, is also closely connected to these areas.

**Conclusion:**

Our investigations demonstrate that the predictiveness curve extended by these new interpretations of areas provides a comprehensive evaluation of markers or sets of markers for prediction.

**Trial registration:**

Austrian Coronavirus Adaptive Clinical Trial (ACOVACT); ClinicalTrials.gov, identifier NCT04351724.

## Background

Predictiveness curves have been proposed to bring together, in a single intuitive graph, the view of the marker’s effect on risk and the view of its classification performance [1]. While the effect can be quantified by, e.g., an odds ratio derived from a logistic regression model, the marker’s importance is better described by measures of predictiveness. This contribution goes beyond known connections between predictiveness curve and explained variation [1] by showing that proportions of areas in the plot of the predictiveness curve can be interpreted as the degrees of necessity and of sufficiency [2].

As an example, consider the study of 578 Covid- 19 patients in the early phase of the pandemic (year 2020, no virus mutations) [3]. The goal was to predict in-hospital mortality after day 4 by laboratory and other factors collected in clinical routine during the first four days in hospital. A cross-validated selection of factors resulted in age, fever on admission, and six variables derived from hematological parameters (mean and slope of lactate dehydrogenase, mean of Creatinine, mean and slope of C-reactive protein, slope of platelet count during the first four days).

**Fig. 1:**
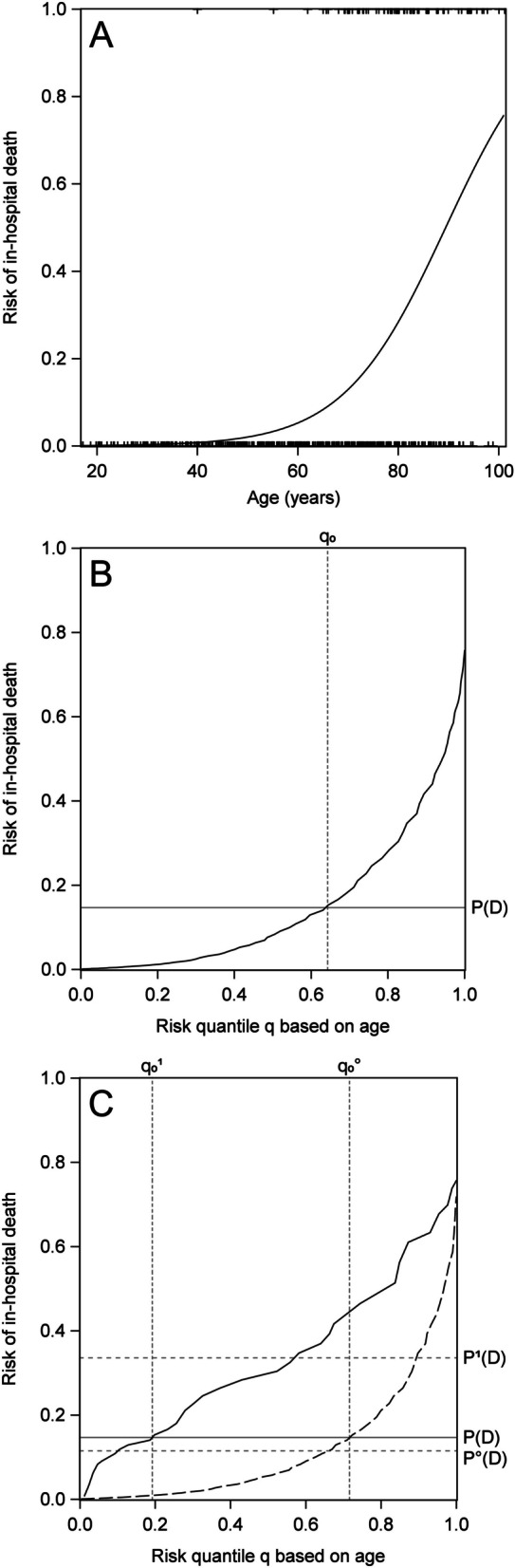
Estimated logistic regression model for predicting in-hospital death after day 4 by age, ticks on bottom and top indicating non-events and events (**A**); smoothed predictiveness curve *R*(*q*) for age on whole sample (**B**), restricted to events (black solid line) and to non-events (black dashed line) (**C**)

Table 1 summarizes various statistical measures estimated from the data using a logistic regression model in SAS 9.4 (SAS Institute Inc., 2020). The odds ratio (OR) for the continuous age variable (1.10 per increase by one year) can hardly be compared to that of the dichotomous presence of fever on admission (OR = 1.83) or to the contribution of the set of six laboratory parameters. In contrast, the proportion of explained variation (EV) allows a clear ranking with EV equal to 0.30, 0.23 and 0.01 for laboratory parameters, age and fever, respectively. A closer look for the risk’s dependence on age is presented in Fig. 1A: While the oldest patients observed a high risk for in-hospital death, this risk is well below the average risk of 14.7% for nearly two thirds of the patients in our sample.

**Table 1:** Marginal (not shrunk) odds ratios (OR) with 95% confidence intervals (CI), apparent marginal proportion of explained variation ($EV_{ind}$), degree of necessity (${DN}_{1}$ and ${DN}_{2}$) and of sufficiency (${DS}_{1}$ and ${DS}_{2}$), Tjur’s coefficient of determination (${C}_{D}$), and standardized total gain ($STG$) for in-hospital death after day 4

Predictor	OR	CI	EV_ind_	DN_1_	DS_1_	DN_2_	DS_2_	C_D_	STG
**Age (years)**	1.10	1.08–1.13	0.23	0.76	0.28	0.70	0.22	0.22	0.53
**Fever on admission**	1.83	1.13–2.97	0.01	0.27	0.04	0.27	0.04	0.01	0.14
**Hematological parameters**^**a**^	-	-	0.30	0.67	0.40	0.62	0.29	0.29	0.53
**Full model**	-	-	0.49	0.88	0.50	0.85	0.40	0.48	0.73

^a^up to day 4 (see text)

The next section summarizes predictiveness curves and their most important features and repeats definitions for measures of predictiveness. Further, the definitions and some important properties of the degree of necessity (DN) and of sufficiency (DS) are collected. "Results" section presents and illustrates novel connections between the predictiveness curve on the one hand, and EV, DN and DS on the other hand.

## Methods

### Predictiveness curves

Predictiveness curves have been proposed as graphical means to integrate the predictiveness of a marker with its performance as a classifier [1]. They show the risk for an event, as derived by a model with one or more predictors, depending on risk quantiles that represent the predictor’s distribution in the underlying population. Figure 1A shows the risk of in-hospital death as predicted by age using a logistic regression model. In the *predictiveness curve* $R(q)$ in Fig. 1B the horizontal axis represents the quantiles $q$ of the predictor distribution. More generally, risk predictions are ordered from lowest to highest values such that the horizontal axis shows quantiles of the risk distribution as predicted by the considered predictor [4].

Figure 1B also indicates the quantile ${q}_{0}$ where the predictiveness curve takes the value of the event prevalence, denoted by $\text{P}(D)$, the probability of the event of interest, $D$. At this point in the risk distribution, prediction conditional on the considered marker(s) equals unconditional risk prediction. The horizontal line at $\text{P}(D)$ also represents prediction using an uninformative marker [1].

**Fig. 2:**
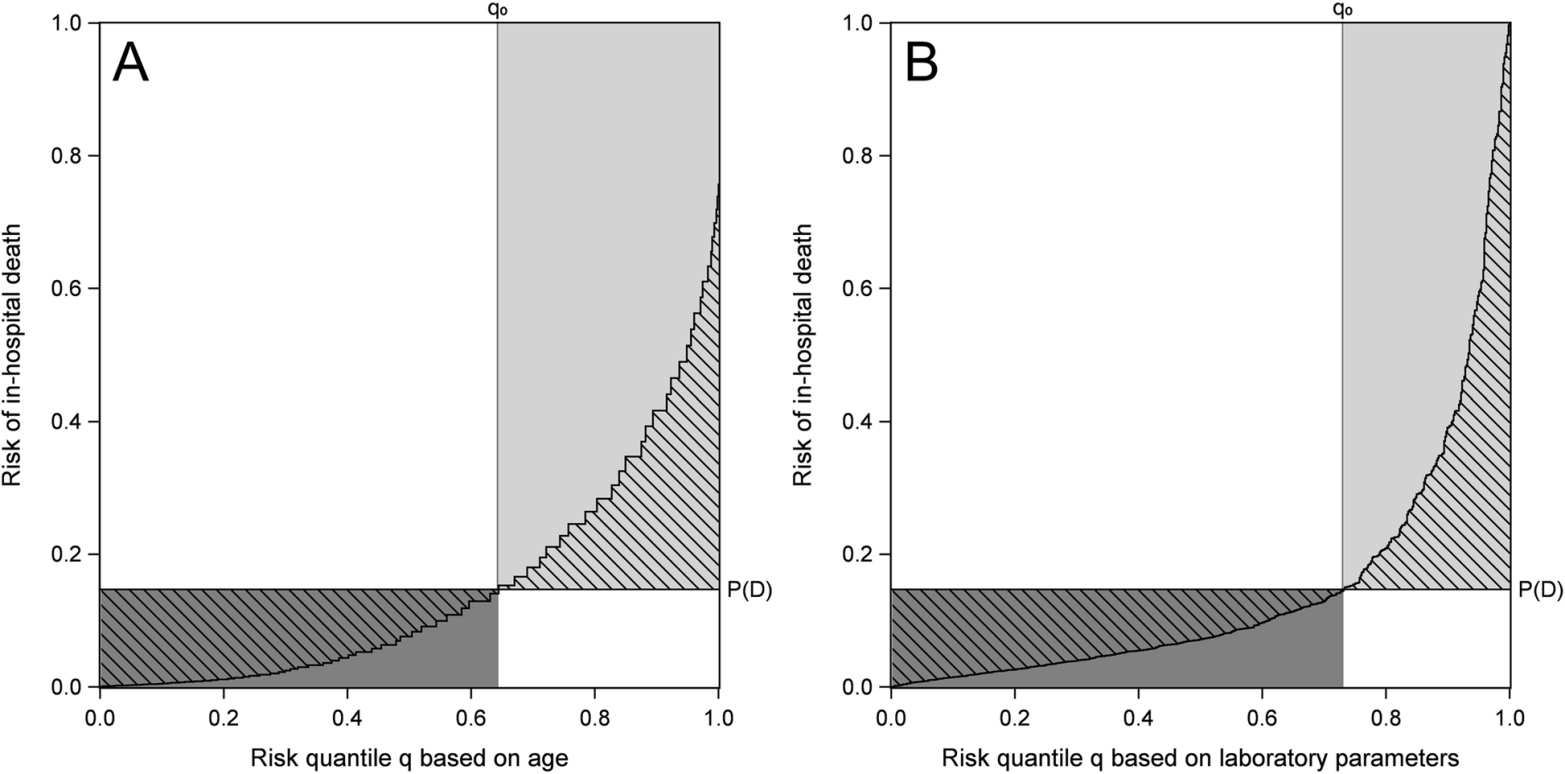
Predictiveness curve with hatched areas ${A}_{N}$ and ${A}_{S}$, for predicting in-hospital death after day 4 by age (**A**) and by laboratory parameters within first four days (**B**)

We denote the geometric areas between $R(q)$ and $\text{P}(D)$ as ${A}_{N}$ (the part below $\text{P}(D)$) and ${A}_{S}$ (the part above $\text{P}(D)$), respectively; they are shown as hatched areas in Fig. 2. Assuming a globally calibrated risk model where conditional risk averages to the unconditional risk in the population, ${A}_{N}$ and ${A}_{S}$ are of equal size [4, 5].

For our example of predicting in-hospital death of Covid- 19 patients after day 4 by age, $\text{P}\left(D\right)=0.147$, ${q}_{0}=0.64$ and ${A}_{N}={A}_{S}=0.066$. The predictiveness curve in Fig. 1B shows that 50% of the patients in our sample have a risk below 8% since $R\left(0.5\right)=0.08$. On the other hand, starting from 30% defined as a risk threshold for in-hospital death, $R\left(0.81\right)=0.3$ shows that 19% of the patients can be regarded to be at high risk.

Figure 3 in Steyerberg et al. [6] shows the predictiveness curve separately for patients without and for patients with the event. This enables to read off, for a given predicted risk level, the resulting specificity and 1 minus sensitivity, respectively, when the considered predictor is dichotomized at the value corresponding to this risk level. For our example data, the predictiveness curve stratified by event status is shown in Fig. 1C. Choosing a risk threshold of 14.7%, equal to unconditional risk, $\text{P}\left(D\right)$, the stratified curves in Fig. 1C cross the threshold at quantile $q_0^0 = 0.72$ and $q_0^1 = 0.19$, respectively, resulting in a specificity of 72% and a sensitivity of 81%.

### Measures of predictiveness

#### Explained variation

The *indirect measure of explained variation* (${EV}_{ind}$) quantifies the relative reduction in predictive inaccuracy when unconditional prediction is replaced by prediction conditional on a considered predictor [7]. EV equals the proportion of variation in the outcome that is explained by the considered predictor(s). It can therefore be used to quantify and compare the importance of prognostic factors [8]. By assuming a globally calibrated model this can be rewritten as the ratio of the variance of conditional predictions to $\text{P}\left(D\right)\left[1-\text{P}(D)\right]$ [2]. Thus, EV involves squared distances between $R(q)$ and $\text{P}\left(D\right)$.

Tjur proposed the *coefficient of discrimination* ${C}_{D}$, a measure of a ‘model’s ability to discriminate between successes and failures’, as the difference of ${\text{P}}^{1}\left(D\right)$ and ${\text{P}}^{0}\left(D\right)$, the expected conditional event probability among events and non-events, respectively [9]. For the example data, ${\text{P}}^{1}\left(D\right)$ and ${\text{P}}^{0}\left(D\right)$ are estimated by the average of the predicted event probabilities across all patients that died in hospital or did not die in hospital, respectively. For age as predictor of in-hospital mortality, ${C}_{D}$ = 0.22 (Table 1) was based on an estimate of 0.331 for ${\text{P}}^{1}\left(D\right)$ and of 0.115 for ${\text{P}}^{0}\left(D\right)$ (see Fig. 1C). By noting that Tjur’s measure ${R}_{mod}^{2}$ exactly equals the plug-in estimator of ${EV}_{ind}$, we may conclude from Proposition 2 in [9] that ${EV}_{ind}={C}_{D}$ (see also [10]). This asymptotic equality is also observed for our example data in Table 1.

#### Total gain

Bura & Gastwirth [5] were the first to propose the (absolute) area between predictiveness curve and $\text{P}\left(D\right)$ as a ‘measure of explanatory power’. They defined *total gain* (TG) as

$$TG={\int }_{0}^{1}\left|\text{P}\left(D\right)-R(q)\right|dq$$

Clearly, $TG={A}_{N}+{A}_{S}$. Since the maximum value of $TG$ is $2\text{P}(D)\left[1-\text{P}(D)\right]$ [5], *standardized total gain* (STG) is defined as

$$STG=\frac{TG}{2\text{P}(D)\left[1-\text{P}(D)\right]}$$

Since the denominator of $STG$ only depends on $\text{P}(D)$, a comparison of $STG$ between different prognostic factors within the same prediction task (and thus with the same event prevalence) is equivalent to a graphical comparison of $TG$. Gu & Pepe [11] have shown that $STG$ equals the *Above average risk difference* (AARD) which is defined as the difference of ${q}_{0}^{0}$ and ${q}_{0}^{1}$, the proportions of conditional predictions below $\text{P}\left(D\right)$ among non-events and events, respectively. AARD also equals Youden’s index evaluated at $\text{P}\left(D\right)$ [11].

### Degrees of necessity and of sufficiency

A cause, represented by certain values of prognostic factors, is considered necessary for an event $D$ if, without the cause, $D$ cannot develop. It is considered sufficient for $D$ if $D$ is unavoidable in the presence of the cause. The degrees of necessity and of sufficiency, ranging from zero to one, have been developed as generalizations of attributable risk based on simple functions of the unconditional event probability $\text{P}\left(D\right)$ and probabilities $\text{P}\left(D|X\right)$ conditional on a predictor $X$ [2]. There are two variants of DN and DS, the first giving more weight to extreme conditional probabilities:

$${DN}_{1}=\sqrt{{\text{E}}_{X<}{\left\{\frac{\text{P}\left(D\right)-\text{P}\left(D|X\right)}{\text{P}\left(D\right)}\right\}}^{2}}$$

$${DS}_{1}=\sqrt{{\text{E}}_{X>}{\left\{\frac{\text{P}\left(D|X\right)-\text{P}\left(D\right)}{1-\text{P}\left(D\right)}\right\}}^{2}}$$

$${DN}_{2}={\text{E}}_{X<}\left\{\frac{\text{P}\left(D\right)-\text{P}\left(D|X\right)}{\text{P}\left(D\right)}\right\}$$

$${DS}_{2}={\text{E}}_{X>}\left\{\frac{\text{P}\left(D|X\right)-\text{P}\left(D\right)}{1-\text{P}\left(D\right)}\right\}$$

where ${\text{E}}_{X<}\left(.\right)$ and ${\text{E}}_{X>}(.)$ denote expectation with respect to $X$ conditional on $\left\{X:\text{P}\left(D|X\right)<\text{P}\left(D\right)\right\}$ and $\left\{X:\text{P}\left(D|X\right)>\text{P}\left(D\right)\right\}$, respectively. For an unfavorable outcome, these two ranges of $X$ values were termed ‘protective’ and ‘harmful’, respectively. Note that ${DN}_{2}$ thus equals the conditional expectation of attributable risk on the protective range [2].

The measures of variant 2 equal weighted differences *between* the event probability on the ‘harmful’ and the ‘protective’ range. In contrast, variant 1 also accounts for the variation of conditional probabilities *within* the ‘protective’ range for ${DN}_{1}$ and *within* the ‘harmful’ range for ${DS}_{1}$ [12]. Therefore, variant 1 is preferred. We have shown that ${DN}_{1}\ge {DN}_{2}$ and ${DS}_{1}\ge {DS}_{2}$ [12].

The product of ${DN}_{2}$ and ${DS}_{2}$ has been shown to provide a lower bound for the proportion of explained variation. The product of ${DN}_{1}$ and ${DS}_{1}$ is generally close to EV with an acceptable discrepancy for practical settings [12].

According to Table 1 none of the predictors or set of predictors up to day 4 is strongly sufficient for in-hospital death of Covid- 19 patients after day 4. This means that on average, the involved independent variables in the specified model do not exhibit a strong ‘force of mortality’ in the ‘harmful’ range. Age is the most important single predictor explaining 23% of the variability of in-hospital mortality, with a high degree of necessity (${DN}_{1} = 0.76$) but low sufficiency (${DS}_{1} = 0.28$). The information derived from repeated measures of four routine laboratory parameters during the first four days explains 30% (with ${DN}_{1} = 0.67$, ${DS}_{1} = 0.40$). As is often the case, EV for the full model (0.49) is limited by the moderate sufficiency of all predictors (${DS}_{1}=0.50$). In contrast, on the necessity side (${DN}_{1}=0.88$), patients with a prognostic index (obtained from the full model) in the ‘protective’ range, may expect an average decrease of in-hospital mortality by 88% relative to unconditional in-hospital mortality.

### Dichotomous markers

In the case of a dichotomous predictor the predictiveness curve is a step function. For fever on admission in the example data the predictiveness curve jumps from the risk of in-hospital death predicted for patients without fever (10.7%) to the risk predicted for those with fever (18.0%) at the risk quantile ${q}_{0}=0.45$ (corresponding to the proportion of patients presenting without fever). Thus, ${A}_{N}$ and ${A}_{S}$ correspond to rectangles with an area of only 0.018. The degrees of necessity and sufficiency are low (Table 1). Note that ${DN}_{1}={DN}_{2}$ and ${DS}_{1}={DS}_{2}$ and ${EV}_{ind}={DN}_{1}\cdot {DS}_{1}={DN}_{2}\cdot {DS}_{2}$ for a single dichotomous prognostic factor [12].

From Section 5 in reference [12] it directly follows that, for locally calibrated models, ${DN}_{2}$ and ${DS}_{2}$ (and by a similar argument $STG$) remain unchanged after dichotomization at the predictor’s average-risk value. In contrast, the preferred measures, ${DN}_{1}$, ${DS}_{1}$ and ${EV}_{ind}$, decrease by an amount that depends on the conditional variances of the risk estimates [12]. These results are confirmed when age is dichotomized at 71 years (corresponding to a risk of $\text{P}\left(D\right)=0.147$) in the data example: ${DN}_{2}=0.71$, ${DS}_{2}=0.22$, $STG=0.54$ (cf. Table 1), while ${EV}_{ind}$ decreases from 0.23 to 0.16, ${DN}_{1}$ from 0.76 to 0.71, and ${DS}_{1}$ from 0.28 to 0.22. This complements warnings against dichotomizing predictors regarding other statistical aspects [13].

## Results

In this section, we show that areas resulting from the predictiveness curve plot are closely connected to EV, DN and DS. Further connections between various measures of prediction performance (including total gain) and their asymptotic properties for hypothesis testing have been studied by Pepe et al. [14].

### DN and DS and the predictiveness curve

In Appendix 1, we show that ${DN}_{2}$ equals the area ${A}_{N}$ between $\text{P}(D)$ and the predictiveness curve for quantiles corresponding to the ‘protective’ range, relative to the total area below $P(D)$ in the same range, which is equal to $\text{P}(D)\cdot {q}_{0}$:

$${DN}_{2}=\frac{{A}_{N}}{\text{P}\left(D\right){q}_{0}}$$

Analogously, ${DS}_{2}$ is equal to the area ${A}_{S}$ between $\text{P}(D)$ and the predictiveness curve on the ‘harmful’ range, relative to the total area above $\text{P}(D)$ in the same range, which is equal to $(1 -\text{ P}(D))\cdot (1 - {q}_{0})$:

$$DS_2=\frac{A_S}{\left(1-\mathrm P\left(D\right)\right)\left(1-q_0\right)}$$

Thus, instead of standardizing $TG={A}_{N}+{A}_{S}$, ${A}_{N}$ and ${A}_{S}$ are each standardized to their respective maximum possible value. Note, however, that these maxima are conditional on ${q}_{0}$ and do not necessarily correspond to a calibrated step function. In contrast, the denominator of $STG$ results from the global calibration constraint leading to a calibrated step function with a step from 0 to 1 at ${q}_{0}=1 -\text{ P}(D)$ [5], in which case ${{DN}_{2}=DS}_{2}$.

Figure 2 shows the numerators of ${DN}_{2}$ and ${DS}_{2}$, ${A}_{N}$ and ${A}_{S}$, respectively, as hatched areas and their denominators as shaded areas. For age ${A}_{N}={A}_{S}=0.0665$ and for the laboratory parameters ${A}_{N}={A}_{S}=0.0660$. This results in nearly identical values for $TG$ as well as for $STG$. However, due to different sizes of the shaded areas for the two predictor sets the values for $DN$, $DS$ and $EV$ differ considerably between these predictors (Table 1). Since ${DN}_{1}\ge {DN}_{2}$ and ${DS}_{1}\ge {DS}_{2}$ these relative areas in the predictiveness plot represent lower bounds for the preferred variant 1 of the degrees of necessity and of sufficiency.

### Explained variation and the predictiveness curve

In Appendix 2 we show that $STG$ is an upper bound for ${EV}_{ind}$. In our example, however, this bound is quite weak (cf. Table 1). Thus, we conclude that ${DN}_{2}\cdot {DS}_{2}\le {EV}_{ind}\le STG$. As explained above, equality for the lower bound holds with a single dichotomous prognostic factor, while for the upper bound with a perfectly separating 0–1 step function at ${q}_{0}=1 -\text{ P}(D)$. Consequently, the proportion of variation in the outcome that is explained by a prognostic marker or a set of markers can be intuitively gauged from the areas ${A}_{N}$ and ${A}_{S}$ produced by the predictiveness curve.

Graphical comparison of predictiveness curves per se between predictors is often hampered by the fact that different predictors imply different values for ${q}_{0}$ (see, e.g., Fig. 2 age vs. laboratory parameters; or Fig. 2 in [1]). In contrast, EV, DN and DS give quantifications of relevant aspects of the predictiveness curve that incorporate ${q}_{0}$. This allows for an easy comparison between predictors, which can be enhanced by a bootstrap-based statistical test [15].

As mentioned in the previous section, ${DN}_{2}$ and ${DS}_{2}$ only quantify the variation of conditional probabilities *between* the mean predictions on the ‘protective’ and the ‘harmful’ range. The same would also hold for any standardization of $TG$ (including $STG$ and a standardization by the sum of the denominators of ${DN}_{2}$ and ${DS}_{2}$). In contrast, ${EV}_{ind}$, ${DN}_{1}$ and ${DS}_{1}$ also account for the variation of conditional probabilities *within* the ‘protective’ range and the ‘harmful’ range [12], making them more sensitive to the shape of the predictiveness curve.

In Fig. 3 of Steyerberg et al. [6] a horizontal line at $\text{P}\left(D\right)$ is shown in a graph of the predictiveness curve stratified by event status. This could be enhanced by horizontal lines at ${\text{P}}^{1}\left(D\right)$ among events and at ${\text{P}}^{0}\left(D\right)$ among non-events, respectively. Their vertical distance thus is an estimate of ${C}_{D}$. For our example data, this is shown in Fig. 1C. Similarly, this plot is enhanced by vertical lines at ${q}_{0}^{1}$ for events and at ${q}_{0}^{0}$ for non-events, with their horizontal distance giving an estimate of $AARD$.

## Discussion

We conclude with some remarks:

Predictiveness curves as well as measures like EV, DN and DS assume a representative sample as their basis. Furthermore, only calibrated models should be compared based on predictiveness curves [16].

In the presentation of the theory and also of the data example, we used apparent values of EV, DN and DS, but also of $R(q)$ (cf. [4]). Since these measures and graphs have been derived on the same data that were used for estimating conditional probabilities bootstrap techniques could be applied to correct for over-optimism.

In the case of non-monotone conditional probabilities, the given definition for $R(q)$ ensures a monotone predictiveness curve by ordering the horizontal axis of the plot according to $\text{P}\left(D|X\right)$ instead of $X$ [4]. Since the definitions of the ‘protective’ and the ‘harmful’ range do not depend on any ordering, the relations presented in this contribution still hold.

Predictiveness curves as well as EV, DN and DS do not depend on the model type used for estimating $\text{P}\left(D|X\right)$. All relations presented here are also valid for estimation algorithms other than the logistic regression model.

Several of the methods presented in this contribution have been extended to survival data, see [2, 7, 17].

SAS and R code for highlighting the areas ${A}_{N}$ and ${A}_{S}$ in the predictiveness curve have been added to the ‘NecSuff’ repository at www.github.com/agleiss.

## Conclusions

This contribution offers an interpretation for the lower and the upper part of the total area between predictiveness curve and the horizontal line representing event prevalence as the degree of necessity and the degree of sufficiency, respectively. In addition, we have shown that certain areas and distances in the plot of the predictiveness curve are connected to measures of predictiveness. Thus, the intuitive approach to compare these features between predictiveness curves for different prognostic factors or sets of factors results in a choice of factors according to their importance.

## Data Availability

The dataset analyzed during the current study is not publicly available but is available from the corresponding author of reference [3] on reasonable request.

## Appendix 1

Denote the predictor’s cumulative distribution function by $F(.)$. By using $x={F}^{-1}(q)$ for a given quantile $q$, $R\left(q\right)=\text{P}\left[D | X={F}^{-1}(q)\right]$ [4]. Since by definition ${q}_{0}=\underset{\text{P}\left(D\right)>\text{P}(D|X)}{{\int }}dF\left(x\right)$ we have

$$\begin{aligned}DN_2=&\left.\mathrm{E}\left[\frac{\mathrm P\left(D\right)-\mathrm{P}\left(D\left|\mathit X\right.\right)}{\mathrm P\left(D\right)}\right|\mathrm P\left(D\right)>\mathrm P\left(D\left|X\right.\right)\right]\\=&\frac1{q_0}\int\limits_{\mathrm P\left(D\right)>\mathrm P\left(D\left|X\right.\right)}\frac{\mathrm P\left(D\right)-\mathrm P\left(D\left|X\right.\right)}{\mathrm P\left(D\right)}dF\left(x\right)\\=&\frac1{\mathrm P\left(D\right)q_0}\overset{q_{0}}{\underset{0}\int}\left[\mathrm{P}\left(D\right)-\mathrm {P}\left(\mathrm{D}\left|F^{-1}\left(q\right)\right.\right)\right]dq\\=&\frac{A_N}{\mathrm P\left(D\right)q_0}\end{aligned}$$

using the substitution $q=F(x)$.

## Appendix 2

Gu & Pepe [11] show that

$${EV}_{ind}={\int }_{0}^{1}\left\{\text{P}\left[\text{P}\left(D|X\right)>r|D\right]-\text{P}\left[\text{P}\left(D|X\right)>r|\neg D\right]\right\}dr$$

with $\neg D$ denoting the counter-event to $D$, and that the integrand is maximized at $r=\text{P}(D)$. Since $AARD$ exactly equals the integrand at $r=\text{P}(D)$ and $STG=AARD$ according to [11], this proves that ${EV}_{ind}\le STG$.

## Abbreviations

AARD: Above average risk difference
DN: Degree of necessity
DS: Degree of sufficiency
EV: Explained variation
OR: Odds ratio
STG: Standardized total gain
TG: Total gain
